# Linear Weak Scalability of Density Functional Theory Calculations without Imposing Electron Localization

**DOI:** 10.1021/acs.jctc.1c00829

**Published:** 2022-03-26

**Authors:** Marcel D. Fabian, Ben Shpiro, Roi Baer

**Affiliations:** Fritz Haber Research Center for Molecular Dynamics and the Institute of Chemistry, The Hebrew University of Jerusalem, Jerusalem 91904, Israel

## Abstract

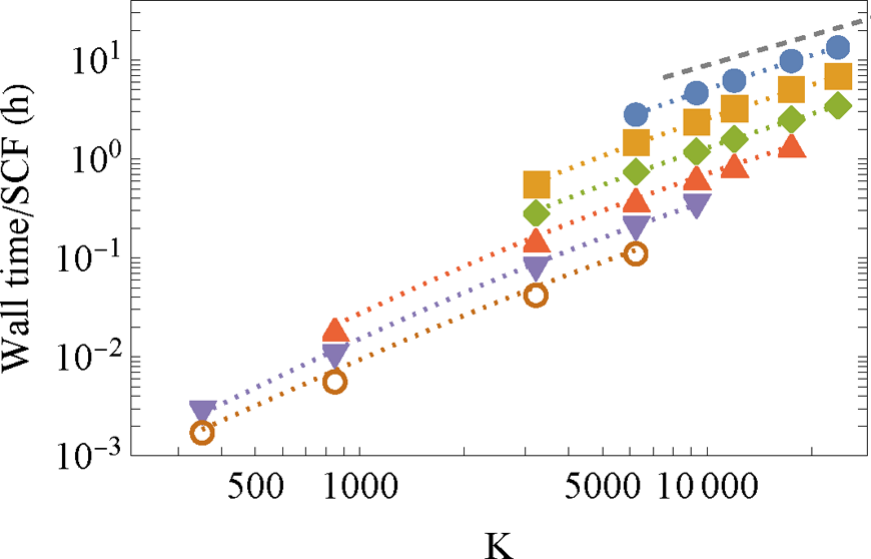

Linear scaling density functional theory (DFT) approaches to the electronic structure of materials are often based on the tendency of electrons to localize in large atomic and molecular systems. However, in many cases of actual interest, such as semiconductor nanocrystals, system sizes can reach a substantial extension before significant electron localization sets in, causing a considerable deviation from linear scaling. Herein, we address this class of systems by developing a massively parallel DFT approach which does not rely on electron localization and is formally quadratic scaling yet enables highly efficient linear wall-time complexity in the weak scalability regime. The method extends from the stochastic DFT approach described in Fabian et al. (WIRES: Comp. Mol. Sci.2019, e1412) but is entirely deterministic. It uses standard quantum chemical atom-centered Gaussian basis sets to represent the electronic wave functions combined with Cartesian real-space grids for some operators and enables a fast solver for the Poisson equation. Our main conclusion is that when a processor-abundant high-performance computing (HPC) infrastructure is available, this type of approach has the potential to allow the study of large systems in regimes where quantum confinement or electron delocalization prevents linear scaling.

## 1 Introduction

In the past few decades, supercomputers’ massive number-crunching power, measured in floating-point operations per second (FLOPS), has grown a million-fold^1^ and is currently pushing toward the exaflop (10^18^ FLOPS) realm. Combining this new technology with electronic structure calculations can revolutionize computational materials science and biochemistry, provided we complement it with algorithms that can efficiently exploit its massively parallel-based infrastructure.

One of the key questions then becomes how to quantify the efficiency of a certain algorithm on a massively parallel machine. A crucial measure in this regard is the *speedup*, which we define as the ratio
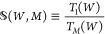
1.1between the wall-times, *T*_1_(*W*), for executing a given computational work *W* using a single processor and *T*_*M*_(*W*) for its execution using *M* processors working in parallel. In operational regimes where the speedup is nearly proportional to *M*, i.e., , there is a clear advantage in using a parallel multiprocessor approach where  is the efficiency, with  being ideal.

The efficient use of parallel computing was discussed by Amdahl in his seminal paper,^2^ in which he identified in *W* an inherently serial (subscript *s*) and parallelizable (subscript *p*) part, *W* = *W*_s_ + *W*_p_. He assumed that the execution wall-time is independent of *M* for completing *W*_*s*_ and decreases linearly with *M* for *W*_p_. Amdahl defined the *serial fraction* as  measured on a single processor machine for a given job independent of *M*. With this definition, the speedup can be expressed as  (*Amdahl’s law*, also called *strong scalability*) and saturates once *M* exceeds the value of 1/*s*_A_.

Gustafson pointed out^3,4^ that in real-world usage the definition for the serial fraction should depend on *M*, because one does not generally take a fixed-sized problem, as Amdahl did, but rather scales the workload *W* with the available computing power. He then defined the serial fraction  as measured on the *M*-processor system and showed that the speedup can be expressed as  (*Gustafson’s law*, also called *weak scalability*), enabling linear speed up which does not inherently saturate as *M* increases.

These considerations can be applied to electronic structure calculations of extended systems in DFT codes that lower the cubic scaling by taking advantage of electron localization.^5−25^ For linear-scaling schemes, the Amdahl serial fraction  is expected to be system-size independent (since both timings in the numerator and the denominator scale linearly with system size) while for codes of higher algorithmic complexity, *s*_*A*_ decreases as system size increases.^26^ In a weak scalability analysis of the linear scaling codes, Gustafson’s serial fraction  is also expected to be system-size-independent (since both timings in the numerator and the denominator scale linearly with system size) and therefore take the form , where *M*_0_ is a constant (depending on the hardware and algorithm). For large *M*, the speedup saturates to , but if *M*_0_ is very large there is a sizable regime where *M* ≪ *M*_0_ and the *s*_*G*_ is essentially zero, so *an ideal linear speedup* emerges, as reported, for example, for the CONQUEST code,^6,27^ even up to *M* = 200 000 cores on the Fujitsu-made K-computer. It is clear from the previous studies mentioned above that it is important to determine the strong and weak scalability properties of codes that can use massively parallel machines, because they are sensitive to many details concerning hardware, system size, algorithmic scaling, etc.

**Figure 1:**
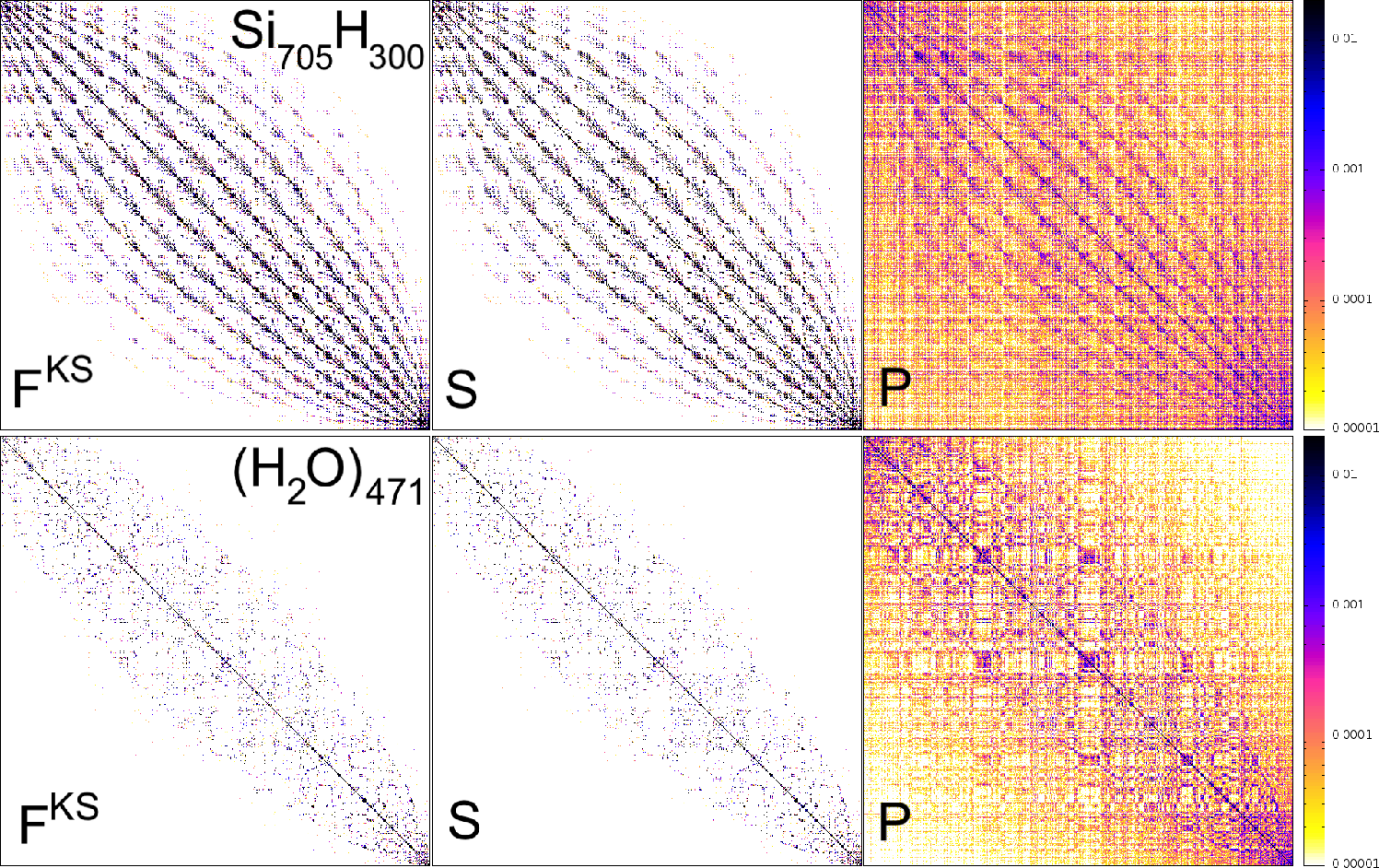
Fock (*F*^KS^), overlap (*S*), and DM (*P*) matrices of Si_705_H_300_ and (H_2_O)_471_ cluster (calculated within the LDA) are shown using a color-coded plot. The basis set in both systems is similar in size, with *K* ≈ 6000. For clearer inspection of the sparsity pattern, the rows and columns are permuted so as to achieve a minimum bandwidth around the diagonal. We applied the MinimumBandWidthOrdering command of Mathematica^34^ to the atomic proximity matrix *D*_*AB*_ = Θ(*R*_0_ – *R*_*AB*_) (where *R*_*AB*_ is the distance between any pair of atoms *A*, *B* and *R*_0_ = 10*a*_0_ is the proximity distance), giving a permutation which is then used to order the atom-centered basis functions.

In this paper we develop an efficiently parallelizable, (semi)local DFT approach which offers quadratic scaling with system size and does not involve approximations derived from assuming electron localization. It combines several approaches, such as atom-centered Gaussian basis sets and real-space grids for providing the electrostatic and exchange–correlation energies (similar to SIESTA^9^ and CP2K/Quickstep^7^) as well as Chebyshev expansion techniques for representing the density matrix.^16,28−30^ We describe the theory and implementation in section 2, where we also provide an illustration of the nonlocalized nature of electrons in the large benchmarking systems we use (see Figure 1). Next, we present the algorithmic complexity and the parallel strong/weak scalability properties of our approach in section 3, and finally, we summarize and discuss the conclusions in section 4.

## 2 Method

In our method, we work with standard quantum chemistry basis sets, composed of atom-centered local functions ϕ_α_(***r***), α = 1, ···, *K*. For calculating the necessary integrals, solving the Poisson equations, and generating the exchange–correlation potentials, we use a 3D Cartesian real-space grid of equidistant points spanning a simulation box, containing the system’s atoms and electronic density. For this purpose, we developed an efficient method for evaluating the basis functions on a relevant set of grid points, outlined in Supporting Information A. Our method of combining basis functions and real-space grids is similar in spirit to those existing in the literature, such as SIESTA^9^ and CP2K/Quickstep,^7^ but differs in important details. Unlike SIESTA, we use standard nonorthogonal Gaussian basis sets, and unlike Quickstep, we represent the basis functions on the grid where all integrals are performed as summations. The first type of integral that we have to evaluate on the grid is the overlap matrix:
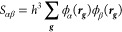
2.1where ***r***_***g***_ are the grid points and *h* is the grid-spacing. Next, the kinetic energy integrals are evaluated as
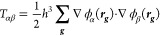
2.2where the derivatives of the basis functions are calculated analytically and then placed on the grid (see Supporting Information A.2.3 for details). To avoid an excessive number of grid points, the equally spaced grid is complemented with norm-conserving pseudopotentials,^31^ representing the effects of the tightly bound core electrons (which are not treated explicitly) and taken into account in the KS Hamiltonian, represented by the Fock matrix
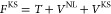
2.3where
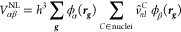
2.4are the integrals for the nonlocal pseudopotential and
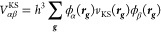
2.5are the KS potential integrals, where
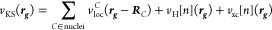
2.6In eq 2.6, *v*_H_[*n*](***r***_***g***_) is the Hartree potential on the grid which is evaluated directly from the grid representation of the electron density *n*(***r***_***g***_) by a reciprocal space-based method for treating long-range interactions.^32^ The exchange–correlation potential *v*_xc_[*n*](***r***_*g*_) (within the local density approximation (LDA)) is also determined on the grid directly from the electron density. From the grid representation of the pseudopotentials^a^ we obtain the potential  appearing in eq 2.6 for nucleus *C* at position ***R***_*C*_ and, by grid integration, the matrix *V*^NL^ appearing in eq 2.3. All integral calculations are performed in parallel for different basis function pairs; for more details, see Supporting Information C.

The electron density on the grid is formally defined as
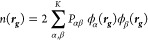
2.7where *P* is the density matrix (DM) and the factor of 2 comes from integration over spin degrees of freedom. The DM must obey an electron-conserving criterion, namely that the integral over all grid points evaluates to the total number of electrons in the system: *h*^3^ ∑_***g***_*n*(***r***_***g***_) = *N*_e_. Indeed, performing this integral and using eqs 2.1 and 2.7 we find
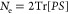
2.8This relation is part of a more general requirement, that the Kohn–Sham eigenstates are populated according to the Fermi–Dirac function *f*_FD_(ε) =  where ε is the corresponding energy eigenvalue. For the DM, this condition can be satisfied by defining^21^
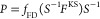
2.9For finite-temperature DFT, β is the inverse temperature and μ is the chemical potential. For ground-state calculations, β obeys β(ε_L_ – ε_H_) ≫ 1, where ε_L_ (ε_H_) is the Kohn–Sham eigenvalue of the lowest unoccupied (highest occupied) molecular orbital. The chemical potential in the Fermi–Dirac function is adjusted to reproduce the systems’ number of electrons *N*_e_ through eq 2.8.

**Figure 2:**
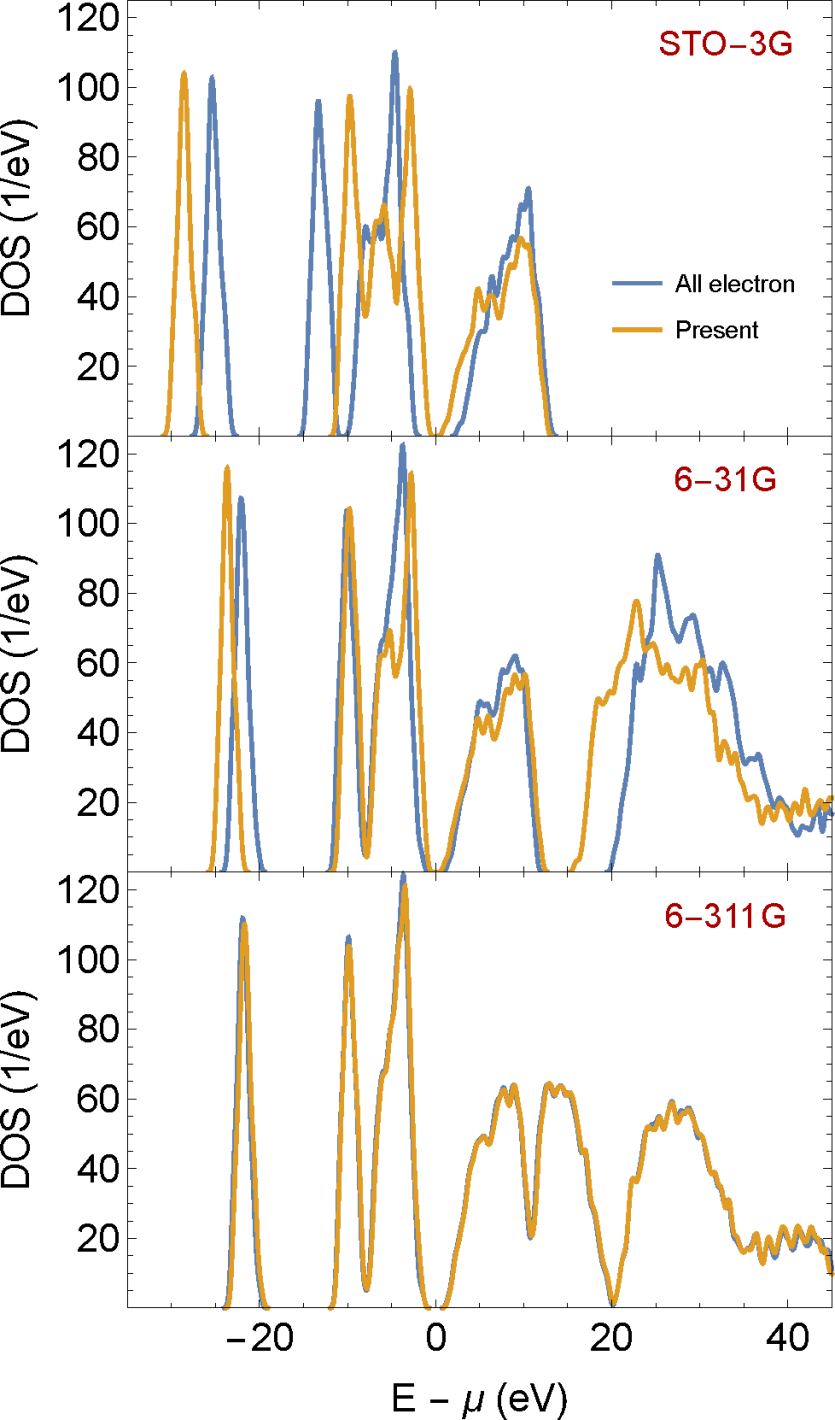
Density of state (DOS) as a function of energy, shifted by the chemical potential μ, for a water cluster (H_2_O)_100_ in the LDA at a fixed geometry. The three panels compare all-electron calculations (performed by Q-Chem^43^) with valence-electron-only calculations (performed by the present approach, using pseudopotentials). Each panel presents the results for different Gaussian basis sets, from single- to triple-ζ quality (STO-3G, 6-31G, and 6-311G). Both calculations use the eigenvalues ε_*n*_ of the converged KS Hamiltonian to obtain the DOS function ρ_DOS_(ε) =  where σ = 0.01*E*_*h*_. The calculation in the present approach used β = 100*E*_*h*_^–1^ with a real-space grid of spacing Δ*x* = 0.33 *a*_0_.

The use of atom-centered local basis functions allows for sparsity in the basic matrices *F*^KS^ and *S*, as illustrated in Figure 1 for two systems of similar size but different chemical nature, a 2.5 nm (diameter) semiconductor nanocrystal Si_705_H_300_ and a 3 nm water cluster (H_2_O)_471_. For the matrix representation in Figure 1, we have ordered the atoms (and the basis functions associated with them) in a way that takes into account their spatial proximity (near atoms tend to have similar indices). Therefore, it is clear by mere inspection that *F*^KS^ and *S* have a relatively small spatial range and are therefore quiet sparse. Our approach makes an effort to exploit this property by using sparse matrix algebra. Despite the spatial locality of *F*^KS^ and *S*, *P* in these large systems is highly nonlocal, expressing the physical fact that the electronic coherence in these systems is long ranged. For the silicon system, this fits our intuition, namely that silicon is by nature a semiconductor, with properties which are close to those of metals. Although water is a large band gap system, it is known that under LDA it exhibits very small HOMO–LUMO gaps^35−38^ (see also Figure 2).

The various expectation values of relevant observables (i.e., operators in the grid representation) can be expressed as trace operations:
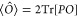
2.10where
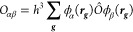
2.11is the matrix representation of the one-body operator *Ô* in the atomic basis. In order to expedite the calculation we need to parallelize the computational work, and this can be done by representing the trace operations as a sum over unit column vectors *u*_α_ (with coordinates (*u*_α_)_β_ = δ_*αβ*_, i.e., zeros in all positions except at α), computed column by column:
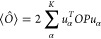
2.12For achieving this, we treat the DM as an operator; that is, we devise a linear-scaling method for applying it to the column vector *u*_α_, based on eq 2.9: *Pu*_α_ = *f*_FD_(*S*^–1^*F*^KS^)*S*^–1^*u*_α_. The operation *S*^–1^*u*_α_ is performed by the linear-scaling preconditioned conjugate-gradient approach involving repeated application of the sparse overlap matrix *S* on column vectors.^b^ The operation of *f*_FD_(*S*^–1^*F*^KS^) on the column vector *S*^–1^*u*_α_ employs a Chebyshev expansion^28,40^ of the function *f*_FD_(ε), which results in repeated applications of the operator *S*^–1^*F*^KS^ to column vectors. Details are described in Supporting Information B. The entire procedure can be readily distributed over several processors in parallel, each commissioned with a distinct set of *u*_α_ column vectors. This calculation method has the additional benefit that it avoids storage of the nonsparse DM. We discuss the algorithmic complexity of the approach, as well as its weak and strong scalability in section 3.

Equations 2.1–2.9 and the techniques of their application discussed above form a series of nonlinear equations that must be solved together, to give the self-consistent-field (SCF) solution. The procedure is iterative and uses the direct inversion of the iterative subspace (DIIS) convergence acceleration method.^41^ Once converged, various expectation values such as charges and multipoles, density of states, and polarizability can be calculated, as well as forces on the nuclei,^42^ which can be used for structure optimization.

In order to check and validate the implementation of the algorithm outlined above, we show in Figure 2 the density of states (DOS) shifted for the chemical potential μ of a cluster of 100 water molecules, obtained with our program, and with the all-electron calculation performed in the commercially available quantum chemistry program Q-Chem.^43^ Our code used β = 100*E*_h_^–1^, but we tested also larger values of β to ascertain that the results are visibly identical. We made comparisons using three different basis sets, ranging from single- to triple-ζ quality (STO-3G, 6-31G, and 6-311G). To complement the picture, we also give the frontier orbital energies, band gaps, and chemical potentials corresponding to these calculations in Table 1.

**Table 1:** Comparison of Frontier Energy Levels, the Band Gap ε_g_ = ε_L_ – ε_H_, and the Chemical Potential μ = (ε_H_ + ε_L_)/2 for the DOS Calculations of Figure 2

basis	method	ε_H_	ε_L_	ε_g_	μ
STO-3G	present	–4.5	–2.6	1.9	–3.6
	all-electron	1.3	4.5	3.1	2.9
	SBKJC	–5.3	–1.6	3.7	–3.4
6-31G	present	–3.5	–1.9	1.5	–2.7
	all-electron	–3.5	–2.2	1.4	–2.8
6-311G	present	–4.7	–3.3	1.4	–4.0
	all-electron	–4.2	–2.7	1.4	–3.4

Looking at the shifted DOS, both the results of Q-Chem and those of the present code converge to indistinguishable values close to that of the all-electron highest-quality basis calculation. This validates our present code’s calculations, even though a small shift still exists between the chemical potentials (−0.6 eV), as seen in Table 1. It is noteworthy that the DOS in our code is less sensitive to basis set quality than the all-electron code, where for the smallest STO-3G basis set the all-electron calculations deviate strongly from the converged basis set values, showing a large (6.3 eV) shift and a band gap which is more than a factor two too large. The stability of our calculations in comparison to Q-Chem can be attributed to the use of the norm-conserving pseudopotentials. Indeed, in Supporting Information F we show that effective core potentials stabilize the Q-Chem small basis set calculations as well.

An additional validation of our approach can be found in the Supporting Information G, where we compare the potential energy surface of the H_2_ molecule calculated with both our code and Q-Chem and where we show the influence of the grid spacing on the accuracy of the calculation. Overall, the approximations that we employ lead to a systematic difference of ∼0.2% in the electronic energy when compared with Q-Chem for most of the examined distance range (and maximally ∼0.4%) and a small corrugation which appears when the gridpoint spacing is larger than the width of the smallest Gaussian primitive. The relative errors in the electronic energy, and the fact that they are mostly a rigid shift, lead to deviance of the order of 0.05 eV in the bond energy, much smaller than typical 6-311G basis set errors.^44^

## 3 Scaling Properties of the Method

In this section we study the method’s algorithmic complexity and analyze the speedup achievable by parallelization in terms of strong and weak scalability.

### 3.1 Algorithmic Complexity

To understand the algorithmic complexity of our method, we have to examine how each part of our code scales as we increase the system size *K*. Here we are especially interested in the asymptotic behavior, meaning that the program part with the largest scaling will determine the overall algorithmic complexity. Our entire SCF cycle, that is described in detail in Supporting Information A.3, includes different integral calculations, solving the Poisson equation, and calculating the density. The integral calculation is expected to scale linearly with system size *K*, i.e. *O*(*K*), because the relevant matrices (*F*^KS^, *S*) are expected to become sparse (see also Figure 1). The Poisson equation is solved by a fast Fourier transform (FFT) which scales as *O*(*N*_*g*_ log *N*_*g*_), where *N*_*g*_ are the grid points, expected to scale linearly with system size. This leaves only the density calculation, which is done according to eq 2.12. The application of the DM *P* to a column vector *u*_α_, expressed through a Chebyshev series, involves repeated applications of the operator *S*^–1^*F*^KS^ to the column vector *v* = *S*^–1^*u*_α_ (see Supporting Information B for details). The length of the Chebyshev expansion, *N*_*C*_, is independent of the system size *K*, and so the algorithmic complexity of the *Pu*_α_ operation is identical to that of one *S*^–1^*F*^KS^*v* operation, namely, linear with *K*. There are a total of *K* different *Pu*_α_ operations (see eq 2.12), so that the overall algorithmic complexity of the method is asymptotically quadratic, i.e., *O*(*K*^2^). As the system size grows our algorithm could be modified to take advantage of the emerging sparsity of the DM, allowing for a *K*-independent complexity of each *Pu*_α_ operation. In such situations, one can expect an overall linear-scaling numerical complexity, i.e., *O*(*K*). However, in the present paper, we focus on the broad class of systems which are very large but for which the DM has not yet localized. Hence, we are in the formally quadratic complexity regime.

**Figure 3:**
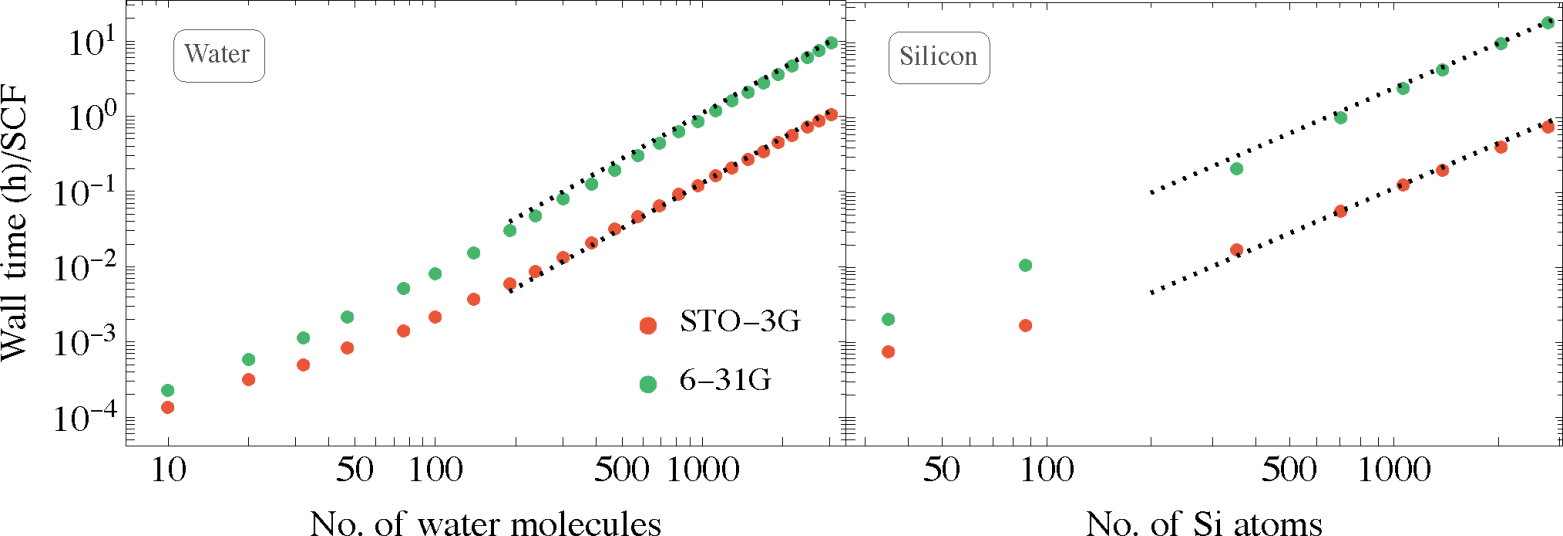
Wall-time as a function of system size, for the water clusters and the silicon nanocrystals, calculated using two basis sets within the LDA. The calculations were performed on eight Intel Xeon Gold 6132 CPU @ 2.60 GHz 755GB RAM (connected through Infiniband), using 112 cores for all systems. The dotted lines in the panels are guides to the eye with designated quadratic scaling. Fitting the function *t* = *Ax*^*n*^ to the data at the larger time range, one obtains the exponent *n* = 1.9 (2.1) for both the water and the silicon systems in the STO-3G (6-31G) basis.

To show that quadratic complexity is indeed what we achieve with this method, we plot in Figure 3 the wall-time per SCF cycle versus system size for water clusters (taken from http://www.ergoscf.org/xyz/h2o.php, accessed on 2022-03-05) and hydrogen-terminated silicon nanocrystals (we use a series of nanocrystals, starting from Si_35_H_36_ reaching Si_2785_H_780_; for details, see Supporting Information D), using STO-3G and the larger 6-31G basis sets. Going from the smaller to the larger basis set increases wall-time by a factor of 10–20. This result is a combination of several characteristics beyond the mere size of the basis set. For example, the magnitude of the Gaussian exponents of the basis set’s primitives are relevant for the dimensioning of the grid. Higher-valued Gaussian exponents require a finer mesh and also increase the kinetic energy component of the Hamiltonian, which increases the Chebyshev expansion length. Smaller (diffuse) Gaussian exponents lead to larger grid windows (see also Supporting Information A.1.1) and hence an increase in overall grid size as well. Furthermore, the implementation of the linear scaling operation of *S*^–1^, involving the incomplete Cholesky decomposition and preconditioned conjugate gradients algorithms, is sensitive to the condition number of *S*, determined by near linear dependencies between basis functions. As seen in the figure, all cases show overall quadratic algorithmic complexity. It is worth noting that the small and intermediate sized systems in the figure exhibit a varying algorithmic complexity with system size associated with the interplay between linear complexity processes having a large prefactor and cubic stages because of the nonsparse nature of the Hamiltonian and overlap matrices.

### 3.2 Strong Scalability

**Figure 4:**
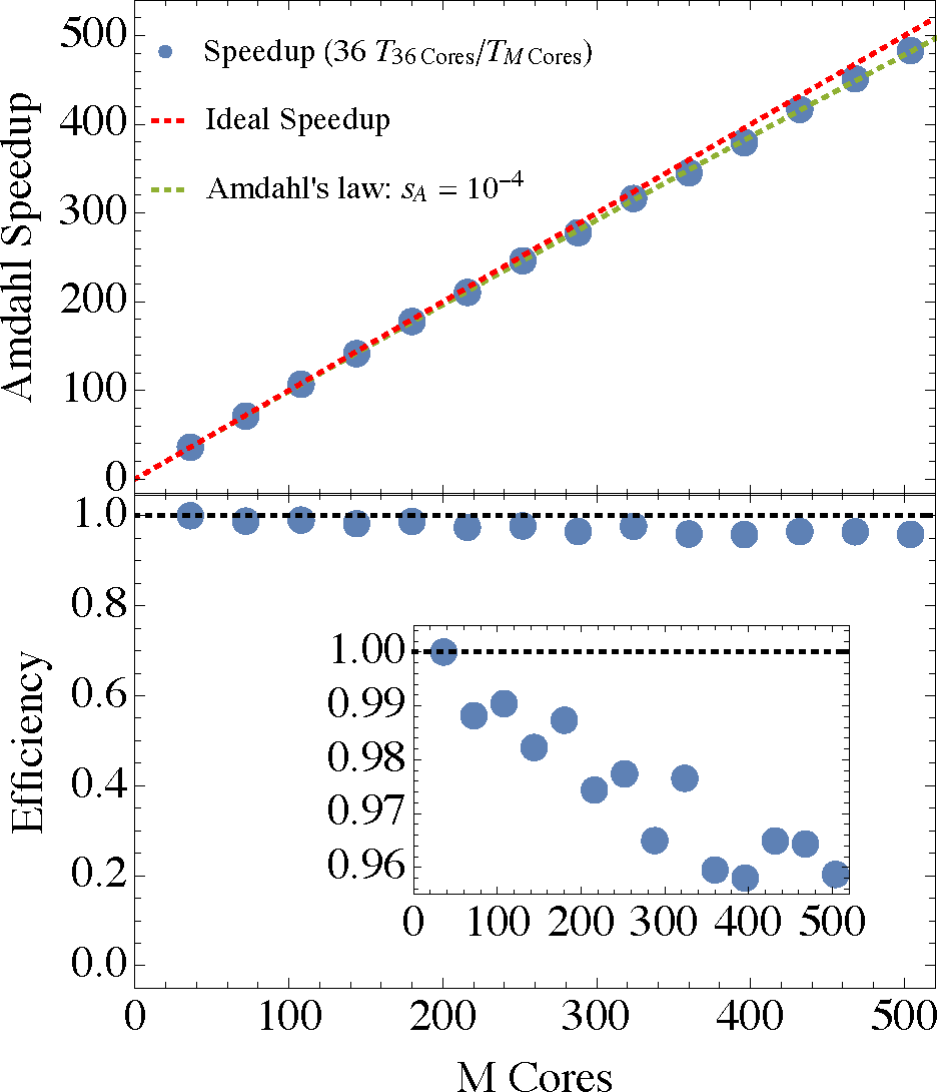
Strong scalability speedup analysis (upper panel) and efficiency  (lower panel) for Si_1379_H_476_. The reference time for 1 processor for the speedup is extrapolated from *T*_1_ = 36*T*_36_. The inset in the lower panel enables a higher resolution of the efficiency regime close to unity. The calculation used the 6-31G basis set (11984 basis functions) within the LDA and were performed on several 2.60 GHz Intel Xeon Gold 6240 with 256 GB using 10 Gb Ethernet networking communications.

In Figure 4 we study the strong scalability properties of our code, i.e., the scalability achievable when increasing the number of processors for a *given* task. We show in the figure the speedup and efficiency for a single SCF iteration of the Si_1379_H_476_ nanocrystal. Our definition for the speedup in eq 1.1 requires the knowledge of the elapsed wall time it takes a single processor (more accurately 1 core) to finish this nanocrystal calculation. Because of (human) time constraints we had to extrapolate this timing from a calculation on 36 cores on one single compute node by *T*_1_ = 36*T*_36_. The results can be analyzed in terms of the Amdahl law finding that the serial fraction is *s*_A_ = 9 × 10^–5^ showing a high degree of parallelization. Accordingly, the parallelization efficiency drops very slowly as the number of processors increases, with 96% efficiency even at *M* = 500 (see the inset in the top panel). We emphasize that this is achieved with a 10 Gb ethernet network communication. Potentially, the decay of efficiency may be slowed down by employing a faster communication solution. According to Amdahl’s law, efficiency will drop to 0.5 when . In Supporting Information E we show results for a smaller system, Si_705_H_300_, where the Amdahl serial fraction is larger, *s*_A_ = 2 × 10^–4^, a system-size dependency due to the quadratic complexity of our method (see our discussion in section 1).

### 3.3 Weak Scalability

**Figure 5:**
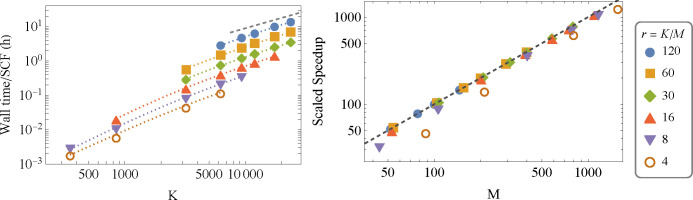
Weak scalability speedup analysis. Left: The wall-time for a single SCF cycle versus the number of basis set functions *K* in calculations given for several fixed values of *r* (*K*/*M* where *M* is the number of processors) on a series of eight hydrogen-terminated silicon nanocrystals (detailed in Supporting Information D) using the 6-31G basis set within the LDA. The black-dashed line is a guide to the eye showing linear scaling wall-time. The calculations were run on several Intel Xeon Gold 6240 CPUs @ 2.60 GHz 256GB RAM connected through a 10 Gb ethernet networking communications. We had access to at most 1584 cores; therefore, for *r* = 4 we could not treat systems greater than *K* = 6420, and similar though less stringent limitations appeared for *r* = 8 and 16. The colored dotted lines are the best-fit of the data to our model in eq 3.4. Right: The scaled speedup as a function of the number of processors *M*, calculated for the six values of *r* from eq 3.2 using the best-fit parameters of our model. The black-dashed line indicates the “perfect” speedup .

In this section we focus on the weak scalability properties of our method, namely how the wall time changes with system size *K* when the number of processors afforded to the calculation *M* grows in fixed proportion *r* = *K*/*M*. In the left panel of Figure 5 we present the wall-time *T* as a function of system size *K* for six series of runs we made with different fixed ratios ranging from *r* = 4 up to *r* = 120 (in the actual calculation, *r* is the number of vectors *u*_α_ assigned to each processor (see eq 2.12)). The markers of each series fall on asymptotically straight lines in the log–log plot which appear parallel to the dark-dashed line, indicating a constant slope of 1. This confirms the claim of achieving linear-scaling wall-time in this regime of operation, where *r* is held constant. We would also like to examine the *speedup* in order to determine the degree of efficiency of our calculation on the parallel machine. For calculating the speedup under our definition in eq 1.1 we need to be able to estimate the wall time *T*_1_(*W*), which for the large systems is not easily accessible because of (human) time constraints. Therefore, we developed the following model for the wall time, with which we will estimate the *M* = 1 wall times:
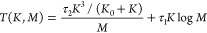
3.1The first term on the right is the dominant parallelizable part of the calculation run on *M* processors (electron density calculation, see Supporting Information C for more information). For *K* ≫ *K*_0_ it exhibits quadratic scaling, while for *K* ≪ *K*_0_ the scaling is cubic because of insufficient sparsity of the Hamiltonian and overlap matrices for small *K*. The second term in eq 3.1 reflects the timing of the serial part of the calculation, dominated by the communication time needed for specific MPI functions (reduce and broadcast) and scales linearly with *K* and logarithmically with *M*.

Using the analytical model, the speedup can now be obtained by plugging eq 3.1 into eq 1.1, resulting in the following closed form expression:
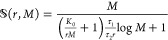
3.2From this equation, it can be seen that for asymptotically large values of *M*, the speedup approaches the limit  and as long as *r* is not too small
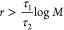
3.3the speedup is close to ideal .

We now fit our model to the calculation’s timing results from the six constant-*r* series shown in the left panel of Figure 5 (a total of 32 data points). This leads to a best-fit set of parameters (in hours): τ_1_ → 5.16 × 10^–6^ h, τ_2_ → 5.63 × 10^–7^ h, and *K*_0_ → 2292.6 for our model, and the resulting fit functions
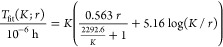
3.4are plotted in the left panel of the figure as dotted colored lines, one for each value of *r*. It can be seen that these fit functions indeed reproduce the actual data (given as points) quite closely.

Having the best-fit parameters, let us now discuss the actual estimated values for the (scaled) speedup in the Gustafson sense. These estimates, based on eq 3.2, are plotted in the right panel of Figure 5. We see that for *r* > 16 the speedup is not too far from ideal, in accordance with the analysis presented above; however, as indicated in eq 3.3, the speedup is smaller when *r* decreases as is clearly visible for *r* = 8 and small *M* and for *r* = 4 for all values of *M*. However, even for these small *r* cases, the speedup is maintained as *M* increases and the calculation is still quite efficient.

## 4 Summary and Conclusions

In this paper we presented a parallelizable electronic structure approach to finite temperature density functional theory under (semi)local functionals using atom-centered Gaussian basis sets which offers linear wall-time complexity as a function of system size in the weak scalability regime. The inherent time complexity of the method is quadratic *O*(*K*^2^), as discussed in section 3.1, and it does not involve truncation of density matrix elements, characteristics of linear-scaling approaches.

Our trace-based calculation combined with Chebyshev expansions allows for efficient parallelization in the strong scalability sense, as shown in subsection 3.2. Because of the quadratic complexity, we found that the value of the Amdahl parameter was system-size dependent, with *s*_A_ = 2 × 10^–4^ for the Si_705_H_300_ system and *s*_A_ = 9 × 10^–5^ for Si_1379_H_476_. The overall weak scalability performance shows that linear scaling wall time is achievable, as demonstrated in section 3.3, and is highly efficient when the number of orbitals per processors *r* is not smaller than ∼10, and beyond that efficiency drops by a factor of ∼1.5.

Our main conclusion is that this type of approach has the potential to be a useful and efficient tool for studying large systems in regimes where quantum confinement or electron delocalization prevents traditional linear-scaling to set in. Furthermore, for even larger systems, where electrons localize, we plan to enable linear scaling either through stochastic orbital methods^21^ or by exploiting directly the DM’s finite range. While in this paper we were concerned mainly with the scalability of the density calculation, force evaluation, done after the density converges, is also an important goal, high on our list of future plans. We will follow our recent work developing a stochastic estimation of the exact energy derivative (Hellman–Feynman) forces.^42^ As shown in ref (45), these stochastic estimations lead to noisy forces that can be used only within Langevin dynamics. In our case, we expect that the deterministic evaluation of the exact derivatives will result in deterministic forces of sufficient quality to enable energy-conserving molecular dynamics simulations.

## Supporting Information Available

Details on our implementation of the Gaussian integral evaluation on the Cartesian grid, the Chebychev expansion, our parallelization strategy, systems studied, density of states calculated with Q-Chem and effective core potentials, and the H_2_ potential energy surface (PDF)

## Notes

Binaries that support the findings of this study are available from the corresponding author upon reasonable request.

## Footnotes

a Here, we use the Kleinman–Bylander (KB) form,^33^ which produces two types of operators, a nonlocal potential operator *v̂*_nl_^*C*^ ≡ *v*_nl_^*C*^(***r*** – ***R***_*C*_, ***r***′ – ***R***_*C*_) which is defined in a small sphere around each atomic core (***R***_*C*_ is the location of atom *C*) and a scalar potential *v*_loc_^*C*^(***r*** – ***R***_*C*_) containing the long-range electron-shielded nucleus Coulomb attraction.

b We use the incomplete Cholesky preconditioning^39^ for the conjugated gradient approach implemented in the HSL-MI28 and MI21 codes, respectively, where HSL is a collection of FORTRAN codes for large-scale scientific computation (http://www.hsl.rl.ac.uk/ accessed 2022-03-05).
