# Supplementary material for: Linear Weak Scalability of Density Functional Theory Calculations without Imposing Electron Localization
Source: J Chem Theory Comput. 2022 Mar 26;18(4):2162–70. doi: 10.1021/acs.jctc.1c00829 (PMC9009081; doi:10.1021/acs.jctc.1c00829)
Supplement: Supplementary file 1 — ct1c00829_si_001.pdf [file ct1c00829_si_001.pdf]

Supporting Information: "Linear weak scalability of density functional theory calculations without imposing electron localization"

A basis set / grid representation

A.1 basis set → grid transformation

Each basis function $\phi_\alpha(\mathbf{r})$ is centered on an atom, located at point $\boldsymbol{R_\alpha}$ and associated with l_α, and m_α, the angular momentum and magnetic quantum number, respectively. The basis functions employed are non-orthogonal Gaussians that are contracted to Gaussian type orbitals (GTOs):

$$\phi_\alpha(\mathbf{r}) = \sum_{p=1}^{N_{cn}} d_\alpha^p \Phi\left(\mathbf{r} - \boldsymbol{R}_\alpha; \zeta_\alpha^p, l_\alpha, m_\alpha\right), \tag{1}$$

where ζ_α^p are the Gaussian exponents, d_α^p are the contraction coefficients N_{cn} is the contraction length, and

$$\Phi(\mathbf{r}; \zeta, l, m) = \sum_{i,j,k} w_{lm}^{i,j,k} \varphi^{ijk}(\boldsymbol{r}; \zeta) \tag{2}$$

are the primitive spherical GTOs which are constructed from the following Cartesian GTOs

$$\varphi^{ijk}(\boldsymbol{r}; \zeta) = x^i e^{-\zeta x^2} \times y^j e^{-\zeta y^2} \times z^k e^{-\zeta z^2}, \tag{3}$$

where $\boldsymbol{r} = (x, y, z)$, $i + j + k = l$ and the weights w^{ijk} are given in Table 1.

In order to evaluate the GTO primitive at a grid-point $\boldsymbol{r}_g = (x_g, y_g, z_g)$, for each Cartesian dimension, e.g. x, we store in memory an array containing, the values of $(x_g - R_{\alpha x})^i \, e^{-\zeta_{\alpha p} \, (x_g - R_{\alpha x})^2}$ (and its derivatives) in the interval $[-L_{\alpha x}, L_{\alpha x}]$ around $R_{\alpha x}$. The memory cost for the three arrays is small since L_α is small due to the compact nature of the Gaussian function. With these considerations, the evaluation of $\varphi^{ijk}(\mathbf{r}_g, \zeta)$ involves just two multiplications, at least an order of magnitude faster than a direct evaluation of the GTO primitive.

Table 1: Cartesian angular momenta and weight derived from the real solid harmonics[1] for $l \leq 2$. Note that only cases where $i + j + k = l$ give non-zero values for d and w.

l	s	p			d							
m	0	1	0	-1	2		1	0			-1	-2
i	0	1	0	0	2	0	1	2	0	0	0	1
j	0	0	0	1	0	2	1	0	2	0	1	0
k	0	0	1	0	0	0	0	0	0	2	1	1
w^{ijk}	1	1	1	1	$\sqrt{3}/2$	$-\sqrt{3}/2$	$\sqrt{3}$	$-1/2$	$-1/2$	1	$\sqrt{3}$	$\sqrt{3}$

A.1.1 The GTO window

The length L_α of the window encompassing the basis function $\phi_\alpha(\boldsymbol{r})$ is determined such that the most protruding primitive GTO has $1 - \eta$ of its "charge" included, where η is a small cutoff parameter. This can be expressed as the solution of the charge equation

$$c\left(L_\alpha \sqrt{\zeta_\alpha}, l_\alpha\right) = 1 - \eta, \tag{4}$$

where $c(d, l) \equiv \frac{\int_0^d x^l e^{-x^2} dx}{\int_0^\infty x^l e^{-x^2} dx}$ and $\zeta_\alpha = \min_p \zeta_\alpha^p$ identify the exponent of the most protruding primitive GTO. An important issue is the choice of η, small values give larger windows, improving accuracy at the expense of a higher computational cost. In Figure 1 the wall-time and accuracy is plotted vs the η parameter and the window length L for a deterministic calculation of $Si_{353}H_{196}$. As η drops from 10^{-3} to 10^{-6}, L grows from 5 to 7 Å (the volume and

number of grid-points grow by a factor of 2.7), the single SCF iteration wall-time increases by a factor of 3, while the relative energy error drops by nearly 3 orders of magnitude.

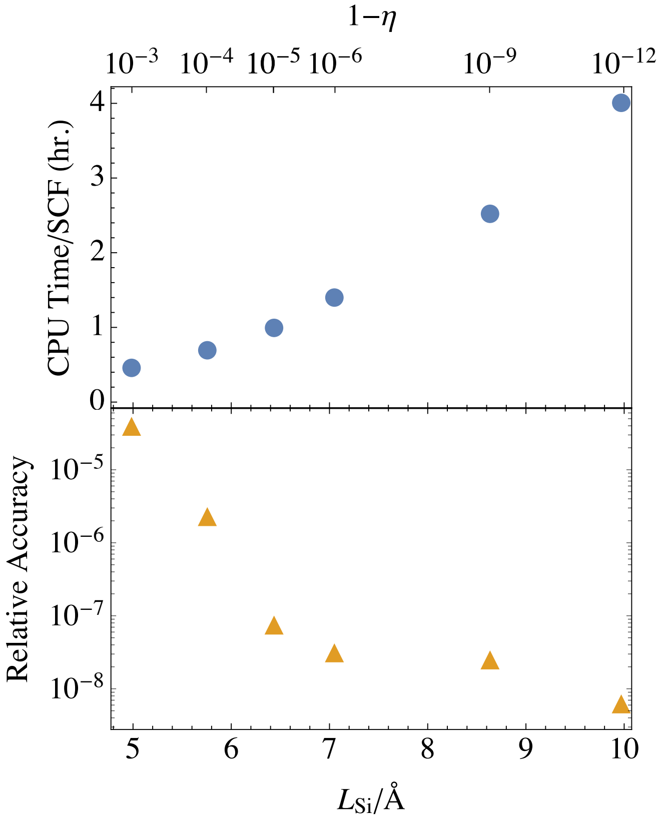

Figure 1: The wall-time of a deterministic LDA calculation (cubic-scaling) of $Si_{353}H_{196}$ and the accuracy in the relative energy vs. η and the silicon atom widow length L_{Si}. The grid spacing was $\Delta h = 0.5a_0$ and the basis set was 6-31G.

Furthermore, not all grid points of the entire grid will be relevant to evaluate the numerical integrals for each pair of basis function. We therefore introduce for each contracted spherical GTO $\phi_\alpha(\mathbf{r}_g)$ a small grid-axes aligned cubic window W_α of lengths $\boldsymbol{L}_\alpha = (L_{\alpha x}, L_{\alpha y}, L_{\alpha z})$, in which the basis function is defined and nonzero. In Figure 2 two such grid windows are displayed for the 2 dimensional case for illustrative purpose (our program uses of course 3D windows): two atomic centers are present (R_α, R_β), each with their own window (W_α, W_β). For each of the windows only a subset of points is relevant and even more so for the overlap of both windows, $\mathrm{W}_{\alpha\beta}$, where only four grid points are relevant in this example.

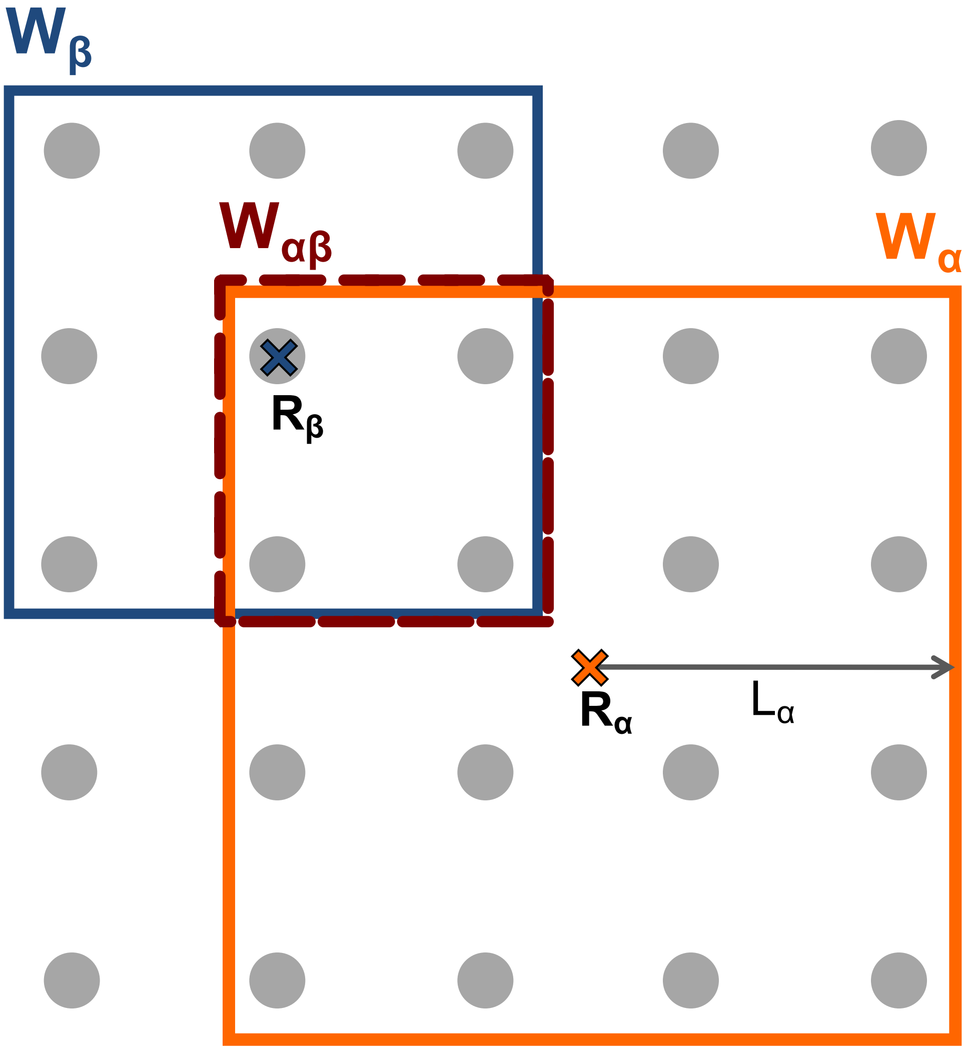

Figure 2: Schematic of grid windows in 2D. Shown are the grid points displayed as grey disks and two atomic centers, R_α and R_β, with their respective GTO windows, W_α and W_β of length L_α and L_β(not shown). The window $W_{\alpha\beta}$ is the region of overlap between W_α and W_β.

A.1.2 The density $n(\boldsymbol{r}_g)$

One important step in the DFT algorithm is to evaluate the electronic density on the grid, based on the density matrix. In order to calculate the electronic density, we define

$$n_\alpha(\mathbf{r}_g) = 2\phi_\alpha(\mathbf{r}_g)\sum_\beta \phi_\beta(\mathbf{r}_g) P_{\beta\alpha} \tag{5}$$

as the density contributed by the α's column of the density matrix P, which can be calculated independently on parallel processors. The calculation is done by the following schematic algorithm:

1. For all $\boldsymbol{r}_g \in W_\alpha$, set $n_\alpha(\boldsymbol{r}_g) = 0$.
2. For each β such that $W_{\alpha\beta} = W_\alpha \cap W_\beta$ (see Figure 2) and $\boldsymbol{r}_g \in W_{\alpha\beta}$, we update the density $n_\alpha(\boldsymbol{r}_g) \leftarrow n_\alpha(\boldsymbol{r}_g) + \phi_\alpha(\mathbf{r}_g)\phi_\beta(\mathbf{r}_g)P_{\beta\alpha}$.

The total density $n(\mathbf{r}) = \sum_\alpha n_\alpha(\boldsymbol{r})$ is then obtained as a reduce operation for all processors. For more details on the parallelization see section C.

A.2 Grid $\rightarrow$ basis set transformation

Here we will explain how to construct the AO matrices from the corresponding grid-operators, the KS potential $v_{KS}(\mathbf{r}) \rightarrow V^{KS}_{\alpha\beta}$, the non-local pseudopotential $\hat{v}_{nl}(\mathbf{r},\mathbf{r}') \rightarrow V^{\mathrm{NL}}_{\alpha\beta}$ and the kinetic energy $-\frac{\hbar^2}{2m_e}\nabla^2 \rightarrow T_{\alpha\beta}$.

A.2.1 KS potential V^{KS} and overlap S

In order to generate a matrix element $V^{KS}_{\alpha\beta}$ in the basis set representation from a grid-based function $v_{KS}(\mathbf{r})$, we only have to sum over the grid points, where both windows W_α and W_β overlap ($W_{\alpha\beta} = W_\alpha \cap W_\beta$, see also Figure 2) :

$$V^{KS}_{\alpha\beta} = \sum_{\mathbf{r}_g \in W_{\alpha\beta}} \phi_\alpha(\mathbf{r}_g)\, v_{KS}(\mathbf{r}_g)\, \phi_\beta(\mathbf{r}_g)\, h^3, \tag{6}$$

with h as the grid spacing. A list is kept in advance for tracking the pairs of orbitals ϕ_α and ϕ_β that actually have a non empty $W_{\alpha\beta}$, expediting the evaluation. The overlap matrix can be calculated this way taking $v_s(\mathbf{r}_g) = 1$ at each gridpoint.

A.2.2 Non-local pseudopotential V^{NL}

While the grid enables a very efficient evaluation of the KS-potential, it is not ideal for describing core electrons. Therefore these electrons are taking into account through norm-conserving pseudopotentials. For atom C at the location $\mathbf{R}_C$, there is, similar to the window for a basis function, a window W^C, i.e. a set of grid points for which the nonlocal part of the pseudopotential, $v_{nl}^{\mathrm{C}}\left(\mathbf{r}_g - \mathbf{R}_C, \mathbf{r}'_g - \mathbf{R}_C\right)$ operates. The matrix V^{NL} representing the non-local part of the pseudopotential in the AO basis has non zero elements $V_{\alpha\beta}^{\mathrm{NL}}$ only for those orbital pairs for which there exists an atom C for which $W_\alpha^C \equiv W^C \cap W_\alpha \neq \emptyset$ and $W_\beta^C \neq \emptyset$ (where $\emptyset$ is the empty set of grid points). In practice, the calculation of V^{NL} is done by locating, for each β all atoms C for which $W_\beta^C \neq \emptyset$ and then calculating the localized function:

$$\psi_{C\beta}(\mathbf{r}_g) = \sum_{\boldsymbol{r}'_g \in W_\beta^C} v_{nl}^{\mathrm{C}}\left(\mathbf{r}_g - \mathbf{R}_C, \mathbf{r}'_g - \mathbf{R}_C\right) \phi_\beta\left(\mathbf{r}'_g\right) h^3, \quad \mathbf{r}_g \in W^C. \tag{7}$$

Then one searches for all orbitals ϕ_α for which $W_\alpha^C \neq \emptyset$ and calculates

$$V_{\alpha\beta}^C = \sum_{\boldsymbol{r}_g \in W_\alpha^C} \phi_\alpha(\mathbf{r}_g)\, \psi_{C\beta}(\mathbf{r}_g)\, h^3 \tag{8}$$

finally, the matrix element is obtained as a sum over all atoms C for which $W_\beta^C \neq \emptyset$:

$$V_{\alpha\beta}^{\mathrm{NL}} = \sum_{C: W_\beta^C \neq \emptyset} V_{\alpha\beta}^C. \tag{9}$$

The pseudo-potential matrix V^{NL} has to be calculated only once in the beginning of the calculation.

A.2.3 Kinetic energy T

The final type of integral considered here is the kinetic energy integral.

$$T_{\alpha\beta} = \frac{1}{2} \sum_{\mathbf{r}_g \in W_{\alpha\beta}} \left[\nabla \phi_\alpha \left(\mathbf{r}_g\right)\right] \cdot \left[\nabla \phi_\beta \left(\mathbf{r}_g\right)\right] h^3. \tag{10}$$

Here we need to take the gradient of the basis function ϕ_α and ϕ_β and multiply them. Taking the gradient of both basis functions rather than the Laplace operator for only one ensures that T will also numerically be positive. For the gradient we need the partial derivatives of the basis functions calculated at each grid point $\mathbf{r_g}$. Currently derivatives up to second order are implemented. They are taken with respect to equation 3.

A.3 Putting everything together: The SCF cycle

In order to solve the non-linear KS equation, one has to do so iteratively, until the SCF solution is found. This process is depicted in Figure 3, with a focus on our unique basis set/grid representation. All integrals are calculated from their respective grid operators, as discussed in section A.2.1-A.2.3. This grid→basis set transformation is depicted in Figure 3 by purple arrows and the corresponding equation number. Except for V^{KS}, all other matrices are only calculated once in the beginning of the SCF cycle. For the start of the SCF cycle the KS-Fock matrix, F^{KS}, is calculated by summing $F^{KS} = T + V^{NL} + V^{KS}$, where the first V^{KS} matrix is determined by an initial guess of the density $n(\mathbf{r})$ (for us usually the sum of atomic densities). The next step in the SCF cycle is to update the density $n(\mathbf{r})$ on the grid. This step is the only basis set→grid transformation and is shown in Figure 3 by a red arrow. As discussed in section A.1.2, the density matrix P is put column-wise on the grid. Each column of P is calculated according to $P = f_{FD}\left(S^{-1}F^{KS}\right)S^{-1}$, where the application of the inverse overlap matrix S^{-1} is handled by a preconditioned conjugate gradient (PCG) approach [a],

[a]We used the incomplete Cholesky preconditioning[2] for the conjugated gradient approach implemented in the HSL-MI28 and MI21 codes, respectively, where HSL is a collection of FORTRAN codes for large scale scientific computation (http://www.hsl.rl.ac.uk/).

thereby only requiring the repeated application of S (which is advantageous, since S is much sparser than S^{-1}) and the matrix exponential ($\exp\left(S^{-1}F^{KS}\right)$) is evaluated by a Chebyshev expansion (see section B). Once the density is calculated, the Hartree potential $v_H(\mathbf{r})$ is obtained by solving Poisson's equation through a fast Fourier transform (FFT)[3] and the exchange-correlation potential $v_{XC}(\mathbf{r})$ by employing the relevant functional form (in our case the local-density approximation (LDA)). The KS potential on the grid $v_{KS}(\mathbf{r})$ is then calculated according to $v_{KS}(\boldsymbol{r_g}) = \sum_{C\in nuclei} v_{loc}^{C}(\boldsymbol{r_g} - \boldsymbol{R}_C) + v_H[n](\boldsymbol{r_g}) + v_{xc}[n](\boldsymbol{r_g})$,where v_{loc} is only calculated once in the beginning of the SCF cycle. The KS potential matrix V^{KS} is updated according to eq. 6 before the next SCF cycle starts with a new KS Fock matrix.

B Chebyshev expansion

The inverse temperature parameter β in the Fermi-Dirac function $f_{FD}(\varepsilon) = \frac{1}{1+e^{\beta(\varepsilon-\mu)}}$ needs to be chosen high enough so that $\beta(\varepsilon_L - \varepsilon_H) \gg 1$ where ε_L (ε_H) is the Kohn-Sham eigenvalue of the lowest unoccupied (highest occupied) molecular orbital. The chemical potential parameter μ must be adjusted to reproduce the number of electrons in the system. For the actual application of the Fermi-Dirac function on a vector u_α we need to evaluate a matrix exponential of a matrix product ($\exp\left(S^{-1}F^{KS}\right)$) and this is achieved by using the Chebyshev polynomial expansion of the Fermi-Dirac function

$$f_{FD}(\varepsilon) = \sum_{l=0}^{N_C-1} a_l(T,\mu)\, T_l\left(\frac{H-\bar{E}}{\Delta E}\right) \tag{11}$$

where $T_l(x)$ are the Chebyshev polynomials obeying the recursion relation $T_{l+1}(x) = 2xT_l(x) - T_{l-1}(x)$, $N_C \approx 2\Delta E\beta$ is the expansion length, and finally: $\Delta E = \frac{E_{max}-E_{min}}{2}$, $\bar{E} = \frac{E_{max}+E_{min}}{2}$, where E_{max} (E_{min}) is the largest (smallest) eigenvalue of $H = S^{-1}F^{KS}$. The Chebyshev expansion coefficients $a_l(T,\mu)$ can be computed using fast Fourier transform methods[4]. The expansion, when applied to the Fermi-Dirac function of the Hamiltonian H allows us to approximate the $\alpha's$ column of the DM $\rho_\alpha \equiv Pu_\alpha$ by the following procedure: First, we set

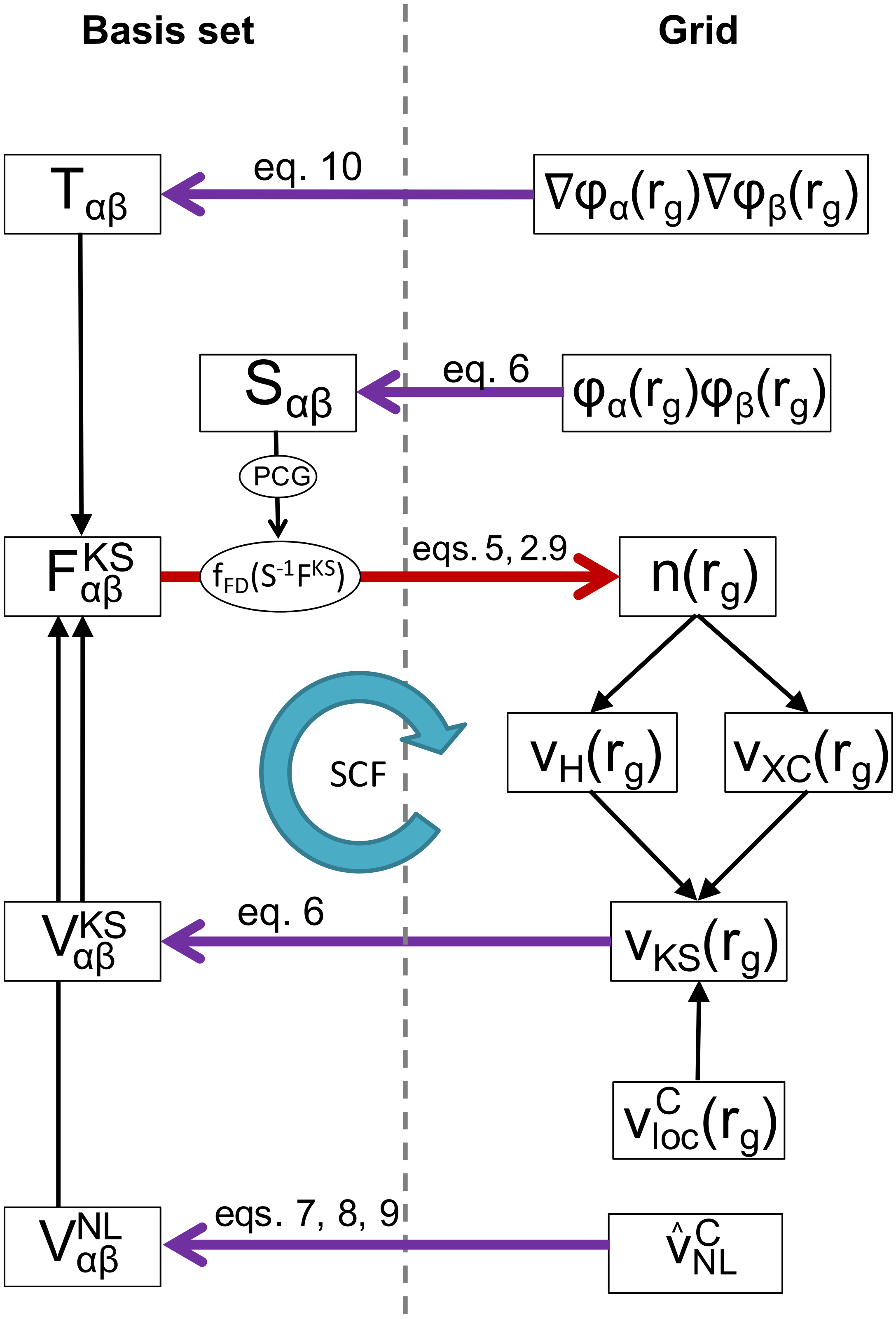

Figure 3: Flowchart for the KS-DFT calculation indicating the stages of the processing and the transformation of data back and forth between the grid and basis set representation during the SCF procedure

$U_0 \leftarrow S^{-1}u_\alpha$, $U_1 \leftarrow H_N U_0$ (where $H_N = \frac{H-\bar{E}}{\Delta E}$ is the "normalized" Hamiltonian) and $l = 2$, then we compute $\rho_\alpha \leftarrow a_0\left(T,\mu\right)U_0 + a_1\left(T,\mu\right)U_1$, and loop until $l = N_C$ the following steps:

$$U_2 \leftarrow 2H_N U_1 - U_0, \tag{12}$$

$$\rho_\alpha \leftarrow \rho_\alpha + a_l(T,\mu)U_2, \tag{13}$$

$$U_0 \leftarrow U_1,\ \ U_1 \leftarrow U_2 \tag{14}$$

$$l \leftarrow l + 1 \tag{15}$$

C Parallelization

In this section we will discuss the main advantage of this algorithm, which is the straightforward path to parallelization. To understand the parallelization strategy a flowchart of the program is depicted in Figure 4. All parallelization shown here is done through the *message passing interface* (MPI).

There are a total number of M processors that can be used and between which the workload in the SCF cycle is split. At first all processors are producing the same initial guess density and an initial KS potential, $n_0(\mathbf{r}_g)$ and $v_{KS_0}(\mathbf{r}_g)$ respectively. This $v_{KS_0}(\mathbf{r}_g)$ is then transferred from the grid to the matrix representation with equation 6. This step is parallelized in such a way that each processor gets a batch of matrix elements $V_{\alpha\beta}^{KS}$ that it needs to calculate and in the end these separate matrix elements are combined and distributed to every processor. The KS-Fock matrix is updated next for every processor, where $F^{KS} = T + V^{NL} + V^{KS}$. T and V^{NL} are calculated once before the SCF cycle according to equation 9 and 10 and are parallelized in the same way as described for V^{KS}. Each processor is independently calculating an electron density on the grid according to equation 5. For this, each processor gets a different set of unit vectors u_α, where the total number of vectors is just the basis set size K. Each processor therefore gets to calculate a fraction $r = {}^{K}\!/\!{}_{M}$

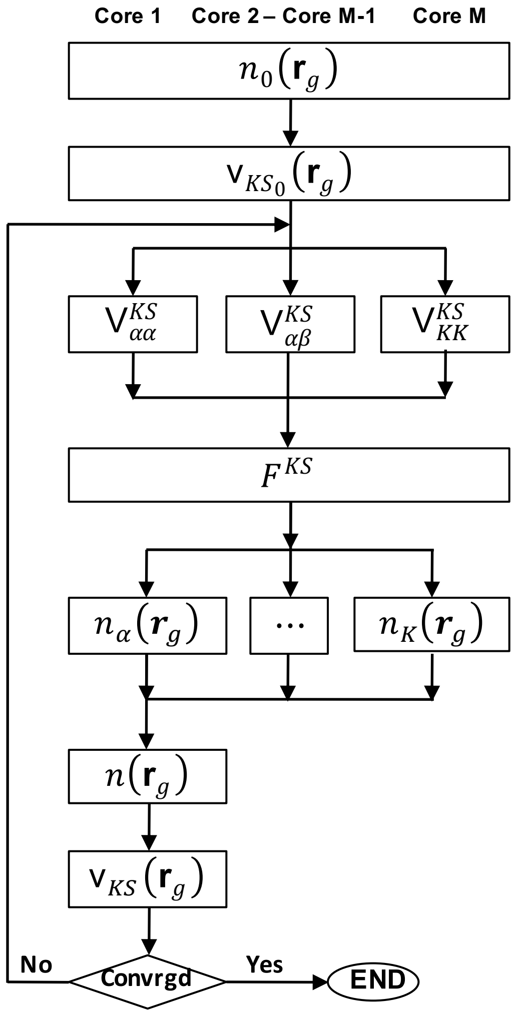

Figure 4: Parallelization strategy for the SCF cycle employed in the program

of the densities. The maximal parallelization that can be achieved in this step is therefore for $M = K$ and $r = 1$. All the densities from the different processors are summed on the first ("master") processor. Only the master process calculates the new potential $v_{KS}(\mathbf{r}_g)$ and checks if the calculation reached the defined convergence criteria ($\Delta E = E^{SCF} - E^{SCF-1}$). If this is the case the calculation finishes, otherwise $v_{KS}(\mathbf{r}_g)$ is distributed to all processors and the SCF cycle is repeated from the calculation of the KS potential matrix. For more details on the SCF cycle see also subsection A.3.

D Systems

Table 2: Silicon clusters employed in the calculations

System	K (STO-3G)	K (6-31G)	$\#e^-$	# atoms
$Si_{35}H_{36}$	176	352	176	71
$Si_{87}H_{76}$	424	848	424	163
$Si_{353}H_{196}$	1608	3216	1608	549
$Si_{705}H_{300}$	3120	6240	3120	1005
$Si_{1063}H_{412}$	4664	9328	4664	1475
$Si_{1379}H_{476}$	5992	11984	5992	1855
$Si_{2031}H_{628}$	8752	17504	8752	2659
$Si_{2785}H_{780}$	11920	23840	11920	3565

Table 3: Water clusters employed in the calculations (taken from `http://www.ergoscf.org/xyz/h2o.php`)

System	K (STO-3G)	K (6-31G)	$\#e^-$	# atoms
$(H_2O)_{10}$	60	120	80	30
$(H_2O)_{20}$	120	240	160	60
$(H_2O)_{32}$	192	384	256	96
$(H_2O)_{47}$	282	564	376	141
$(H_2O)_{76}$	456	912	608	228
$(H_2O)_{100}$	600	1200	800	300
$(H_2O)_{139}$	834	1668	1112	417
$(H_2O)_{190}$	1140	2280	1520	570
$(H_2O)_{237}$	1422	2844	1896	711
$(H_2O)_{301}$	1806	3612	2408	903
$(H_2O)_{384}$	2304	4608	3072	1152
$(H_2O)_{471}$	2826	5652	3768	1413
$(H_2O)_{573}$	3438	6876	4584	1719
$(H_2O)_{692}$	4152	8304	5536	2076
$(H_2O)_{816}$	4896	9792	6528	2448
$(H_2O)_{964}$	5784	11568	7712	2892
$(H_2O)_{11120}$	6720	13440	8960	3360
$(H_2O)_{1293}$	7758	15516	10344	3879
$(H_2O)_{1481}$	8886	17772	11848	4443
$(H_2O)_{1698}$	10188	20376	13584	5094
$(H_2O)_{1924}$	11544	23088	15392	5772
$(H_2O)_{2165}$	12990	25980	17320	6492
$(H_2O)_{2469}$	14814	29628	19752	7407
$(H_2O)_{2737}$	16422	32844	21896	8211
$(H_2O)_{3050}$	18300	36600	24400	9150

E Strong scalability $Si_{705}H_{300}$

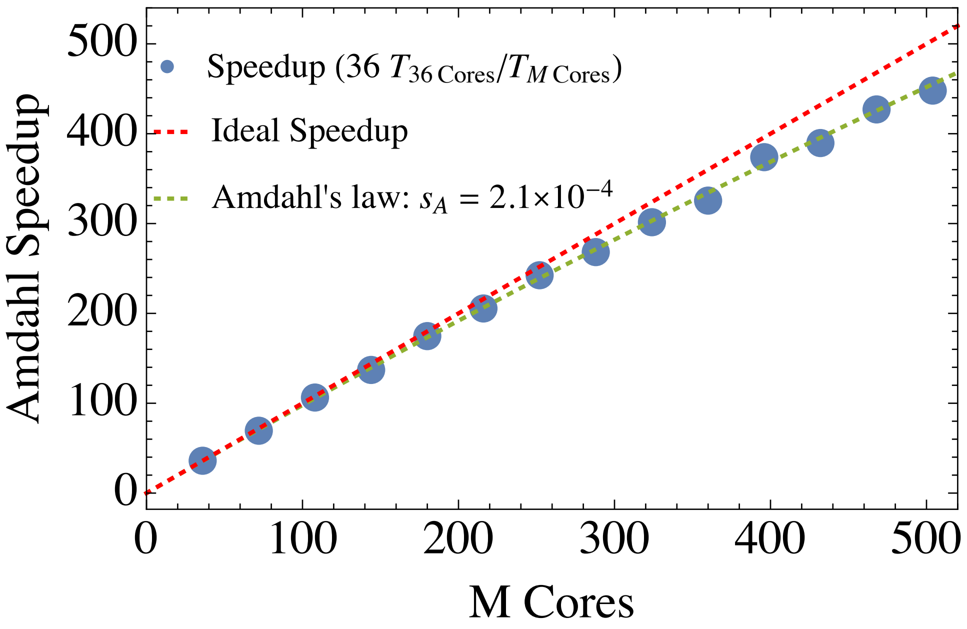

Figure 5: Strong scalability speedup analysis for $Si_{705}H_{300}$. The reference time for 1 processor for the speedup is extrapolated from $T_1 = 36T_{36}$. The calculations used the 6-31G basis set (6240 basis functions) within the LDA and were performed on several 2.60GHz Intel Xeon Gold 6240 with 256 GB using 10Gb Ethernet networking communications.

F DOS $(H_2O)_{100}$

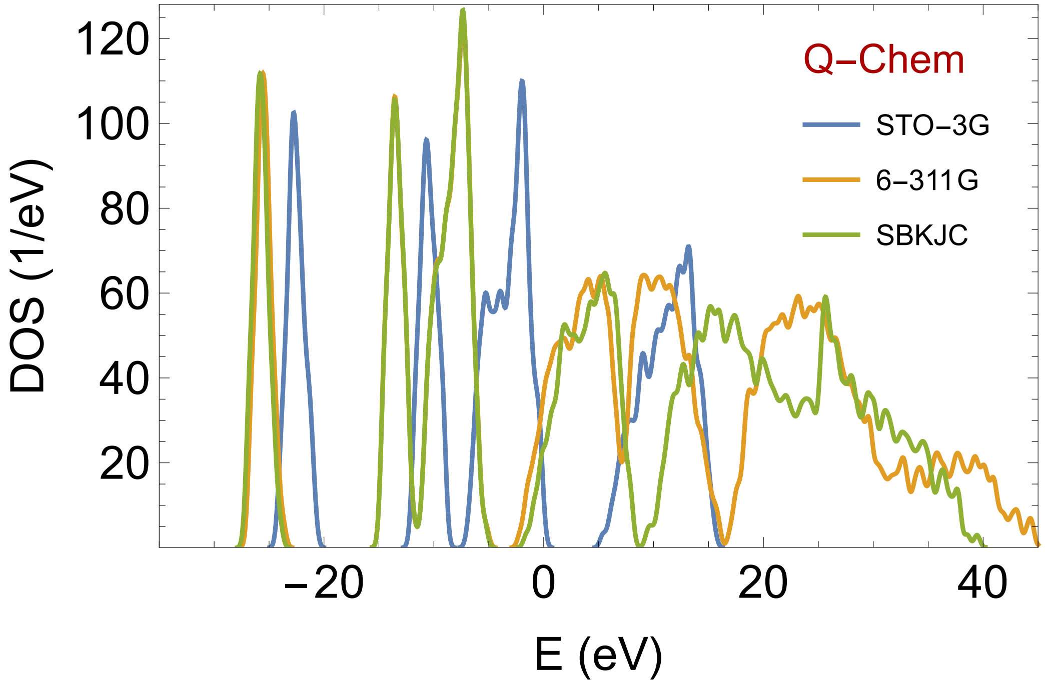

Figure 6: The DOS as a function of energy for a water cluster $(H_2O)_{100}$ using the Q-CHEM[5] program for an all-electron LDA calculation with the STO-3G and 6-311G basis set (same data as in Figure 2.2 in the main paper, but not shifted) and for the standard SBKJC[6] effective core potential (ECP) in Q-Chem, which uses a close to minimal valence basis set. The DOS are plotted using $k_B T = 0.01 E_h$.

In Figure 2.2 of the main paper we show that for the STO-3G basis set our code gives a better discription of the density of states (DOS) in the band gap region compared to the all-electron calculation of Q-Chem. We attribute this to the fact that our code uses norm-conserving pseudo-potentials. In order to check this point we ran Q-Chem with the effective core potential (ECP) SBKJC[6], which uses a close-to-minimal basis set and the results are given in figure 6. It can be seen that similar to the norm-conserving pseudo-potential calculation of our code, the band gap of the SBKJC ECP calculation is closer to the larger 6-311G basis set result. Furthermore, it is noteworthy that the DOS in the occupied states offered by SBKJC is nearly identical to that of the 6-311G all-electron calculation.

G H_2 potential energy surface

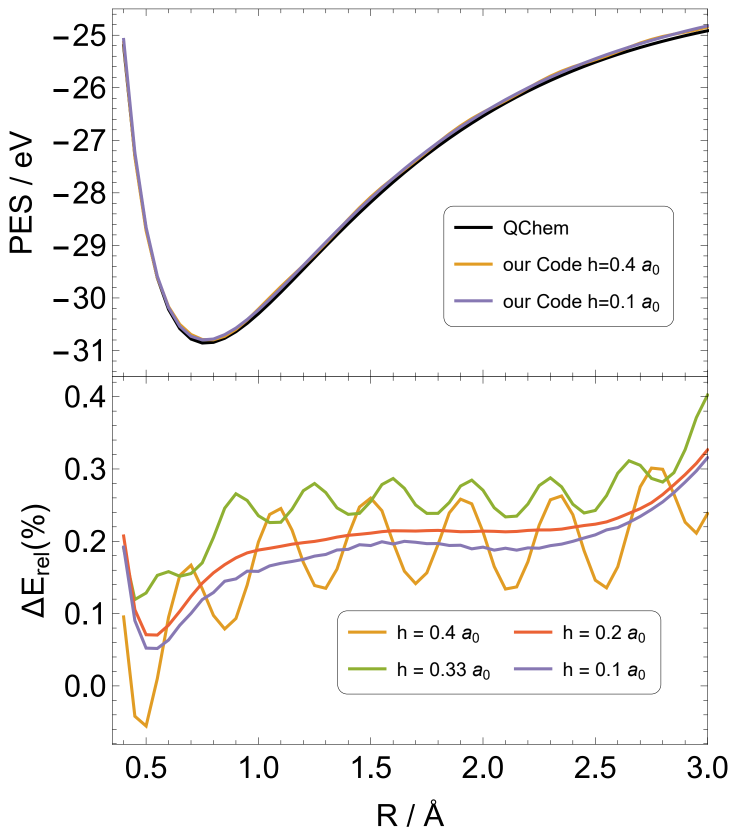

Figure 7: Upper panel: The H_2 molecule potential energy surface (PES) $V_{BO}(R) = E_{elec}(R) + \frac{e^2}{4\pi\epsilon_0}\frac{1}{R}$, where R is the distance between the nuclei and $E_{elec}(R)$ is the DFT electronic energy. We show results calculated with our program (for different values of the grid point spacing h) and with Q-Chem. Lower panel: The relative electronic energy differences (in percent) $\Delta E_{rel} = 100 \times \frac{E_{elec}^{our} - E_{elec}^{QChem}}{\left|E_{elec}^{QChem}\right|}$.

The potential energy surface (PES) is calculated in the upper panel of Figure 7 for the hydrogen molecule with the 6-311G basis set and the local density approximation (using the PW92 parametrization[7]) for our code and for Q-Chem. For our code we chose the following additional parameters:

- grid length L: $32a_0$ in every cartesian direction, chosen large enough with $L_{\zeta_{min}}\sqrt{\zeta_{min}} \gg$

1 (see eq. (4), here $\zeta_{min} \approx 0.1 a_0^{-2}$ from the 6-311G basis set) to include the basis functions at all distances.

- $\eta = 10^{-12}$ (see eq. 4)
- $\beta = \infty$ (matrix diagonalization instead of chebychev expansion for the density matrix P)

For the Q-Chem calculation we chose the following specific keywords (additional to the basis set and functional selection):

- xc_grid 3
- thresh 14

Comparing our results with Q-Chem in the upper panel of Figure 7 we have overall a good agreement in absolute energies. In the lower panel we show the PES differences between Q-Chem and our code for different values of grid point spacing h. It is noticeable that there are oscillations, also called grid corrugations, that decrease with the grid spacing parameter h. This is due to the fact, that we use the 6-311G basis set in these calculations for which the highest Gaussian exponent is $\zeta_{max} \approx 34 a_0^{-2}$. In general we must have $h\sqrt{\zeta_{max}} \ll 1$, so that in our case $h \ll 0.17\, a_0$; indeed, as h increases beyond this value the corrugations increase as well. There is also a small positive shift in all our energy calculations compared to Q-Chem's, except for short molecular distances where there are smaller and bigger differences. Both the shift and the sensitivity at the short bond length can be attributed to the pseudopotentials.

Overall the approximations that we employ compared with Q-Chem's lead to a systematic difference of $\sim 0.2\%$ in the electronic energy for most of the examined distance range (and maximally $\sim 0.4\%$) and a corrugation which can be suppressed by taking smaller grid point spacing.

References

(1) Helgaker, T.; Jorgensen, P.; Olsen, J. *Molecular Electronic-Structure Theory*; Wiley, 2014.

(2) Scott, J.; Tůma, M. HSL_MI28: An Efficient and Robust Limited-Memory Incomplete Cholesky Factorization Code. *ACM Trans. Math. Softw.* **2014**, *40*, 1–19.

(3) Martyna, G. J.; Tuckerman, M. E. A reciprocal space based method for treating long range interactions in ab initio and force-field-based calculations in clusters. *J. Chem. Phys.* **1999**, *110*, 2810–2821.

(4) Baer, R.; Head-Gordon, M. Chebyshev expansion methods for electronic structure calculations on large molecular systems. *The Journal of Chemical Physics* **1997**, *107*, 10003–10013.

(5) Shao, Y. et al. Advances in molecular quantum chemistry contained in the Q-Chem 4 program package. *Mol. Phys.* **2015**, *113*, 184–215.

(6) Stevens, W. J.; Basch, H.; Krauss, M. Compact effective potentials and efficient shared-exponent basis sets for the first- and second-row atoms. *The Journal of Chemical Physics* **1984**, *81*, 6026–6033.

(7) Perdew, J. P.; Wang, Y. Accurate and simple analytic representation of the electron-gas correlation energy. *Phys. Rev. B* **1992**, *45*, 13244–13249.
